# Synergistic Effect of Flavonoids and Metformin on Protection of the Methylglyoxal-Induced Damage in PC-12 Neuroblastoma Cells: Structure–Activity Relationship and Potential Target

**DOI:** 10.3390/molecules29102306

**Published:** 2024-05-14

**Authors:** Danyang Zhang, Xiaoshi He, Ting Wang, Yan Xing, Zhilong Xiu, Yongming Bao, Yuesheng Dong

**Affiliations:** 1 MOE Key Laboratory of Bio-Intelligent Manufacturing, School of Bioengineering, Dalian University of Technology, Dalian 116024, China; zhangdanyang96@mail.dlut.edu.cn (D.Z.); docxyxy@163.com (Y.X.); zhlxiu@dlut.edu.cn (Z.X.); biosci@dlut.edu.cn (Y.B.); 2 Department of Endocrinology, The Second Hospital of Hebei Medical University, Shijiazhuang 050004, China; 15512157887@163.com; 3 School of Ocean Science and Technology, Dalian University of Technology, Panjin 124221, China

**Keywords:** flavonoids, metformin, synergistic neuroprotection, structure–activity relationship, ROS mediation, apoptosis, signal pathway related to Aβ formation

## Abstract

Methylglyoxal-induced ROS elevation is the primary cause of neuronal damage. Metformin is a traditional hypoglycemic drug that has been reported to be beneficial to the nervous system. In this study, flavonoids were found to enhance the protective effect of metformin when added at a molar concentration of 0.5%. The structure–activity relationship (SAR) analysis indicated that ortho- substitution in the B ring, and the absence of double bonds between the 2 and 3 position combined with the gallate substitution with R configuration at the 3 position in the C ring played crucial roles in the synergistic effects, which could be beneficial for designing a combination of the compounds. Additionally, the mechanism study revealed that a typical flavonoid, EGCG, enhanced ROS scavenging and anti-apoptotic ability via the BCL2/Bax/Cyto C/Caspase-3 pathway, and synergistically inhibited the expression of GSK-3β, BACE-1, and APP in PC-12 cells when used in combination with metformin. The dose of metformin used in the combination was only 1/4 of the conventional dose when used alone. These results suggested that ROS-mediated apoptosis and the pathways related to amyloid plaques (Aβ) formation can be the targets for the synergistic neuroprotective effects of flavonoids and metformin.

## 1. Introduction

Alzheimer’s disease (AD) is a chronic neurodegenerative disorder characterized by neuronal damage. Methylglyoxal (MG), has demonstrated neurotoxicity and is implicated in the presence of amyloid plaques (Aβ), a well-established pathological hypothesis underlying AD [1]. It has been shown that MG levels in AD patients are twice as high as those in the controls [2]. MG is a critical biomarker of the early diagnosis of AD for early intervention [3]. In recent years, it has been proven that AD and diabetes mellitus (DM) share the same pathological features [4]. For this reason, AD is referred to as type 3 diabetes (T3D) [5]. Diabetic patients have a higher risk of developing AD [6] and cognitive impairment is also a complication. MG is a by-product of glycolysis that accumulates when diabetic patients experience hyperglycemia [7], and its level is reported to increase two- to four-fold under these conditions [8].

The mechanism study indicated that the accumulation of MG contributes to oxidative stress resulting from excessive reactive oxygen species (ROS) and further leads to cell apoptosis, exacerbating neurodegeneration and neuronal apoptosis [9]. Oxidative stress and cellular damage caused by MG have also been reported in neuron-like PC12 cells [10], Furthermore, the cognitive impairment driven by diabetes in PC-12 cells has been considered an effective model to evaluate the pharmaceutical effects and elucidate the mechanism of the drug or natural products [11].

Metformin (*N*,*N*-dimethylbiguanide) is a biguanide antidiabetic drug recommended as first-line treatment for diabetic patients and taken orally [12]. In recent years, it has been shown to be beneficial to the nervous system [13]. A prospective observational study observed that metformin slowed the development of cognitive decline in diabetic patients aged 70–90 years [14]. Similar studies have been reported by other groups. For example, metformin was reported to relieve cognitive decline in Aβ-injured rats by reducing neuroinflammation and oxidative stress [15]. It also mitigated cognitive dysfunction by suppressing mitochondrial fission in diabetic mice [16]. Thus, some researchers have considered that improving cognitive impairment might be a new indication for metformin in the future [17]. The best clinical dosage of metformin is as high as 2000 mg/d; however, the risk of lactic acidosis in patients with kidney disease is increased by taking metformin [18], which has a high mortality rate. Thus, in order to utilize metformin in clinical treatment to improve cognitive impairment, its efficacy needs to be improved and toxicity needs to be reduced. A recent study indicated that natural products were comparable or even superior to metformin at lower dosages. For example, a mixture of *Sclerocarya birrea*, *Nauclea latifolia*, and *Piper longum* extracts [19], puerarin [20] et al. have suggested that combining the clinical drugs with natural products can enhance its efficacy of clinical drugs by extending the simple mechanism of its activity. A study has shown that *portulaca oleracea* L. enhanced the anti-inflammatory, antioxidant, and autophagic effects of metformin in diabetic rats [21]. It also found that gallic acid enhanced the antioxidant, anti-apoptosis, and anti-inflammatory effects in rats with acute hepatic encephalopathy [22]. Hence, a combination of metformin with natural products might be an effective means of enhancing the efficacy and reducing the toxicity for improving the cognitive impairment.

Flavonoids are a family of plant-derived compounds with pharmacological activity. A previous study by our group reported that flavonoids and acarbose synergistically inhibited α-glucosidase [23]. We have also reported that flavonoids could alleviate MG-induced damage in PC-12 cells [24]. However, to the best of our knowledge, there are no reports on the role of natural products, including flavonoids, in enhancing the neurological benefits of metformin and reducing its toxicity. The present study aims to investigate the synergistic protective effects of metformin and flavonoids on the protective effects against MG-induced damage in PC-12 cells, while also analyzing their structure–activity relationship (SAR). This research endeavors to provide an alternative approach for the treatment or amelioration of neurodegenerative diseases. The SAR data could also provide a theoretical basis for the design of the combination. The mechanism underlying the synergistic protective effect on neuronal cells was further investigated to facilitate the discovery of novel therapeutic agents.

## 2. Results

### 2.1. Combined Effect on Cell Viability

**Figure 1:**
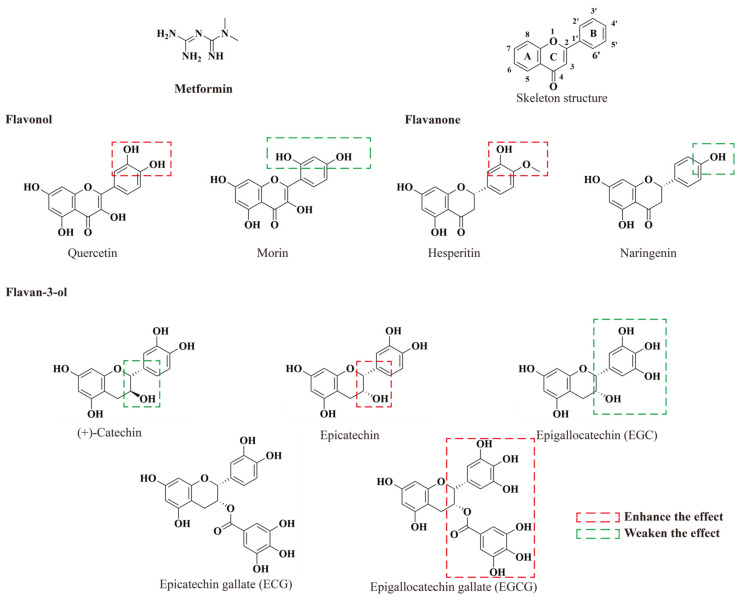
Structure of flavonoids tested and metformin.

In our previous study, 25 flavonoids were tested, and 8 of them showed a protective effect against MG-induced damage in PC-12 cells [24]. The active flavonoids included the flavonols, quercetin (Q), and morin (M), the flavanones, hesperetin (H), and naringenin (N), and the flavan-3-ols, Epicatechin (EC), Epigallocatechin (EGC), Epicatechin gallate (ECG) and Epigallocatechin gallate (EGCG), whose structures were summarized in Figure 1. Furthermore, (+)-catechin (C) was also selected as the control. To examine the cellular toxicity, PC-12 cells were treated with nine flavonoids (3.13−25 μM) and metformin (0.625–5 mM) at various concentrations for 48 h. The CCK-8 assay showed that flavonoids and metformin concentrations from 1.56 to 25 μM, and concentrations from 0.313 to 5 mM, respectively, showed no toxicity (Figure S1). Furthermore, the cellular toxicity of each flavonoid of diverse concentrations in combination with metformin was also tested by CCK-8 assay (Figure S1). Compounds combinations with no cytotoxicity were employed in the treatment groups in the following study.

### 2.2. Combined Effect of MG-Induced PC-12 Cells by Flavonoids and Metformin

**Figure 2:**
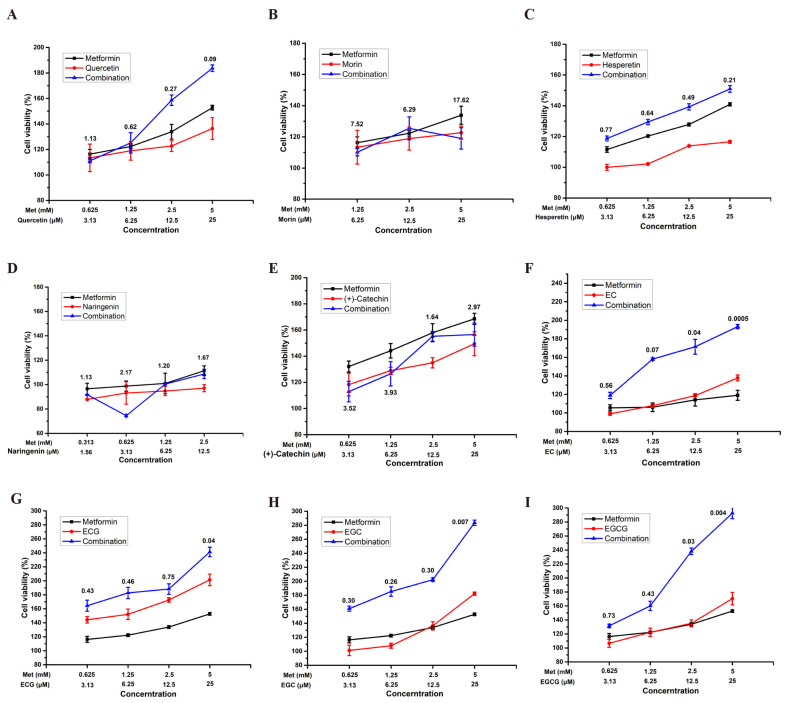
Protective effect of the combination of metformin and (**A**) quercetin, (**B**) morin, (**C**) hesperetin, (**D**) naringenin, (**E**) (+)-catechin, (**F**) EC, (**G**) ECG, (**H**) EGC, (**I**) EGCG against MG-induced PC-12 cells. CI values above the data points were calculated according Chou and Talalay’s method. CI < 0.9, CI = 0.9−1.1, and CI > 1.1 indicate synergism, additive effect, and antagonism, respectively. The experiments were performed in triplicate.

The protective effect of the combinations of active flavonoids and metformin was tested by CCK-8 assay in PC-12 neuroblastoma cells. The combination index (CI) value based on Chou−Talalay Method [25] was selected to evaluate the synergistic effects, and the results are shown in Figure 2.

In general, flavonoids with hydroxyl or methoxy substitution at the ortho position of the B ring showed a more pronounced synergistic effect when they combined with metformin at a metformin/flavonoid molar ratio of 200:1. Examples of such flavonoids included quercetin, hesperetin, EC, EGC, ECG, and EGCG. Flavonoids without a hydroxyl or methoxy substitution at the ortho position on the B ring, such as morin and naringenin, did not show synergistic effects, and even showed antagonistic effects.

Comparing the CI values of quercetin, hesperetin and EC revealed that the ortho-substitution in the B ring, as well as the absence of a double bond between the 2 and 3 positions, and gallate substitution with R configuration at the 3 position in the C ring, play considerable roles in enhancing the synergistic effect. The concentration of the synergistic combination of quercetin was significantly higher than that of hesperetin. Hesperetin produced a synergistic effect with metformin at a concentration of only 3.13 μM, while the concentration of quercetin that showed a synergistic effect was one time higher. EGC and EGCG, which lacked double bonds at the 2 and 3 positions, displayed a good synergistic effect at relatively low concentrations with metformin.

Comparing the CI values of (+)-catechin and EC also revealed that the substitution at the 3 position was crucial for the synergistic effect. The flavan-3-ols with an R configuration at the 3 position, such as EC, EGC, ECG, and EGCG, all showed good synergistic effects with metformin, while (+)-catechin with an S configuration at the 3 position did not show any synergistic effect and even exhibited a strong antagonistic effect.

The presence of an R configuration of gallic acid substitution at the 3 position on the C ring also influenced the synergistic effect. When there was lack of an R configuration of gallic acid substitution at the 3 position on the C ring, the more ortho-hydroxyl was substituted on the B ring, the weaker the synergistic effect was. The typical example was the combination of EC and metformin, whose CI value was generally lower than that of the combination of EGC and metformin, except for the lowest concentration combination (0.625 mM metformin and 3.13 μM EC). However, when an R configuration of gallic acid was introduced at the 3 position on the C ring, the greater ortho-hydroxyl substitution on the B ring enhanced the synergistic effect. The combination of ECG and metformin has a generally lower CI value than the EGCG and metformin combination, except for at the lowest concentration combination (0.625 mM metformin and 3.13 μM ECG).

The protective effects of the combination against methylglyoxal-induced damage were examined in human neuroblastoma SH-SY5Y cells. Specifically, the synergistic interaction of EGCG and metformin on MG-induced SH-SY5Y cells damage was investigated. The results demonstrated a synergistic effect with CI values ranging from 0.008 to 0.84, with a molar ratio of metformin to EGCG of 100:1.

### 2.3. The Mechanism of Combination of EGCG and Metformin on the Protection of MG-Induced PC-12 Cells

As EGCG expressed all the characteristics of synergistic effects (ortho-substitution in the B ring, the absence of double bonds between the 2 and 3 positions, gallate with an R configuration substitution in the 3 position on the C ring) in the SAR analysis, and its combination with metformin showed relatively stronger synergistic effects on the cell viability of MG-induced PC-12 cells damage, therefore, it was selected in the following study to explore the mechanism of synergistic effect.

#### 2.3.1. The Effects of the Combination on ROS Generation in MG-Induced PC-12 Cells

**Figure 3:**
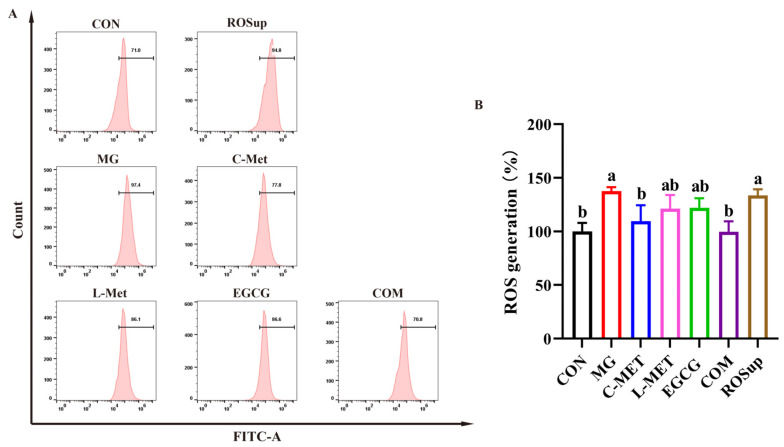
Effects of the combination of metformin and EGCG against MG-induced PC-12 cells’ ROS generation. MG: 0.5 mM. C-Met: 10 mM. L-Met: 2.5 mM. EGCG: 12.5 μM. COM: 2.5 mM Met + 12.5 μM EGCG. ROSup: the positive control. (**A**) Analysis of ROS generation by flow cytometry. (**B**) Statistical analysis of the ROS fluorescence intensity. The data shown are the mean ± SD of three independent experiments. Different letters indicate significant differences between the two groups. (*p* < 0.05).

The production of intracellular ROS was examined through flow cytometry using a DCFH-DA probe. As shown in Figure 3, the ROS accumulation was increased by 137% of the CON group (regarded as 100%) in PC-12 cells induced by 0.5 mM MG for 48 h and 133.52% when PC-12 cells were treated with 0.5 mM MG for 48 h and ROSup, respectively, which were both significantly higher compared to the CON group (regarded as 100%) (*p* < 0.05). The ROS accumulation in the L-Met group and the EGCG group showed a recovery of 121.27% and 121.97%, respectively, although these changes were not statistically significant compared to the MG group (*p* > 0.05). Notably, the intracellular ROS level was alleviated to the normal level in the COM group (99.72%), which was not significantly different compared to the CON group (*p* > 0.05).

#### 2.3.2. The Effects of Combination on Cell Apoptosis in MG-Induced PC-12 Cells

**Figure 4:**
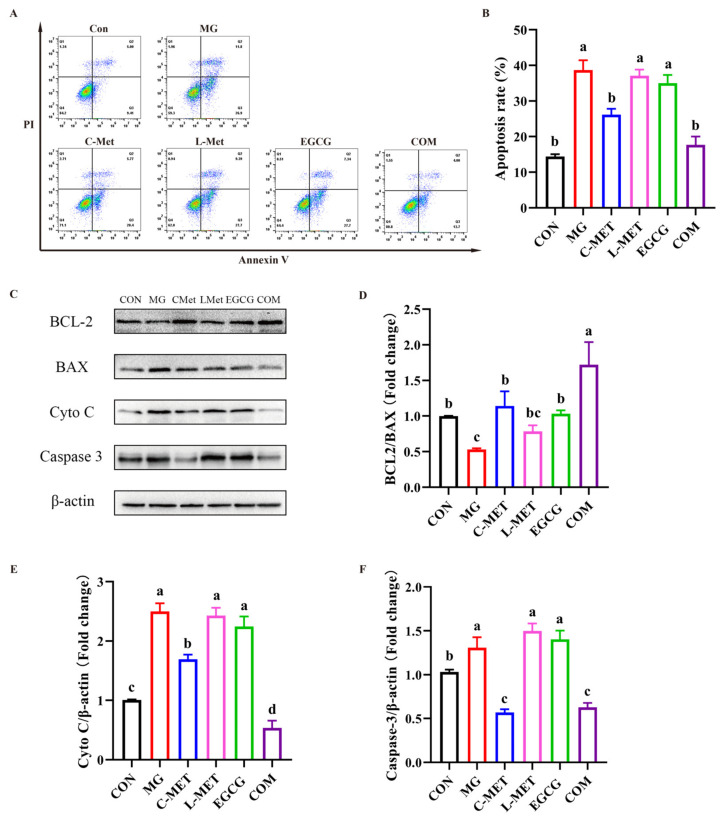
Effects of the combination of metformin and EGCG against MG-induced PC-12 cells’ apoptosis. MG: 0.5 mM. C-Met: 10 mM. L-Met: 2.5 mM. EGCG: 12.5 μM. COM: 2.5 mM Met + 12.5 μM EGCG. (**A**) Analysis of cell apoptosis by flow cytometry. (**B**) Statistical analysis of the apoptosis rate. (**C**) Western blot results of the expression levels of apoptosis proteins. Statistical analysis of the expression levels of the ratio of Bcl2 and Bax (**D**), cytochrome C (Cyto C) (**E**), and Caspase-3 (**F**). The data shown are the mean ± SD of three independent experiments. Different letters indicate significant differences between the two groups. (*p* < 0.05).

The effects of the combination on cell apoptosis in MG-induced PC-12 cells were detected using flow cytometry. As shown in Figure 4A,B, cell apoptosis was induced in PC-12 cells after being treated with MG for 48 h. The early apoptosis (Q3) rate and late apoptosis (Q2) rate increased to 26.9% and 11.8%, respectively, resulting in a total apoptosis rate of 38.7%. This increase was found to be statistically significant when compared to the CON group (*p* < 0.05). Additionally, the results showed no effects on cell apoptosis in either the EGCG group or the L-Met group, with no significant difference compared with the MG group. (*p* > 0.05). However, cell apoptosis was relieved in MG-induced PC-12 cells in both the C-Met group and the combination group after treatment for 48 h in MG-induced PC-12 cells. The combination group exhibited significantly lower levels of both early and late apoptosis compared to the MG group (*p* < 0.05), which indicated that EGCG enhanced the antiapoptotic effect of metformin in MG-induced PC-12 cells. It should be noted that to reach the similar antiapoptotic effects, with no significance between the C-Met group and COM group (*p* > 0.05), the dose of metformin in the combination was only 1/4 of its conventional dose (10 mM) when used alone.

Relevant proteins associated with apoptosis were subsequently assessed via Western blot analysis. As shown in Figure 4C–F, after being treated with MG for 48 h, the expression of BCL2/BAX decreased and the expression of cytochrome C (Cyto C) and caspase-3 increased significantly compared to the CON group (*p* < 0.05). In the C-Met group, BCL2/BAX was elevated and the expression of Cyto C and caspase-3 was inhibited significantly compared to the MG group (*p* < 0.05). Moreover, the expression of BCL2/BAX was significantly higher in the COM group compared to both the MG group and the groups treated with L-Met or EGCG individually. However, neither L-Met nor EGCG treatment alone had a significant effect on the expression of Cyto C and caspase-3 when compared to the MG group (*p* > 0.05). In contrast, the COM group exhibited a significant decrease in the expression of Cyto C and caspase-3 compared to the MG group (*p* < 0.05).

#### 2.3.3. The Regulation of the Combination on Pathway Related to Aβ Formation

**Figure 5:**
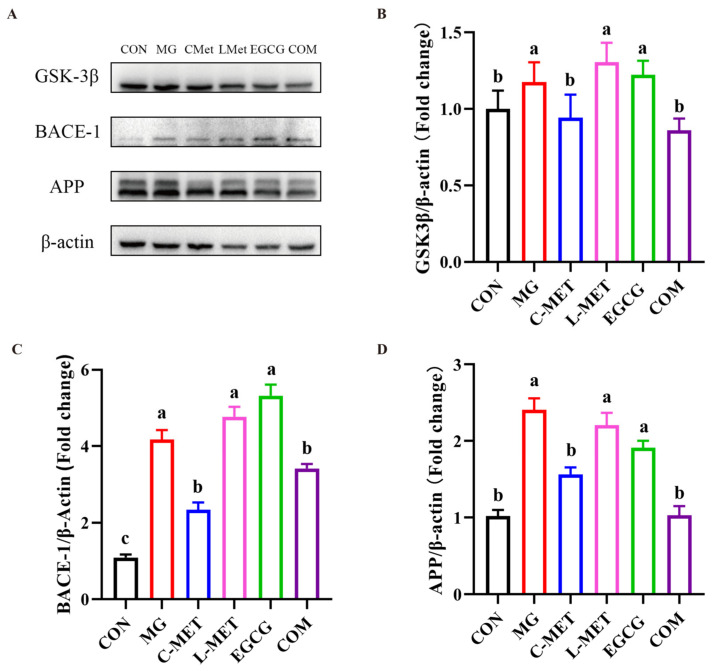
Effects of the combination of metformin and EGCG on GSK 3β/BACE1/APP against MG-induced PC-12 cells. MG: 0.5 mM. C-Met: 10 mM. L-Met: 2.5 mM. EGCG: 12.5 μM. COM: 2.5 mM Met + 12.5 μM EGCG. (**A**) Western blot results of the expression levels. Statistical analysis of the expression levels of GSK 3β (**B**), BACE-1 (**C**), and APP (**D**). Data shown are the mean ± SD of three independent experiments. Different letters indicate significant differences between the two groups. (*p* < 0.05).

Subsequently, the synergistic effects of this combination on the expression of GSK-3β, BACE-1, and APP, which are closely related to Aβ formation [26], were evaluated using Western blot to elucidate the mechanism of cell protection. The results demonstrated a significant increase in GSK-3β in the MG group compared to the CON group of PC-12 cells (*p* < 0.05). Treatment with metformin improved this situation, and the expression of GSK-3β was decreased in a dose-dependent manner. The conventional dose of metformin (10 mM) significantly reduced the expression of GSK-3β (*p* < 0.05), while treatment with a low dose of metformin (2.5 mM) or EGCG alone had no effect on the expression of GSK-3β, which did not differ significantly compared to that of the MG-group (*p* > 0.05). When MG-induced PC-12 cells were treated with the COM group, the expression of GSK-3β was decreased significantly compared to the MG group (*p* < 0.05), and showed no significant difference when compared to the group that received the conventional dose of metformin (*p* > 0.05). A similar phenomenon was also observed in the regulation of the expression of BACE-1 and APP. The results indicated that the combination of metformin and EGCG protected PC-12 cells by regulating GSK-3β/BACE-1/APP, and its effect was comparable to that of the conventional dose of metformin (Figure 5A–D).

#### 2.3.4. Combination of EGCG and Metformin Inhibited Apoptosis and Pathway Related to Aβ Formation of MG-Induced PC-12 Cells through Suppressing ROS

**Figure 6:**
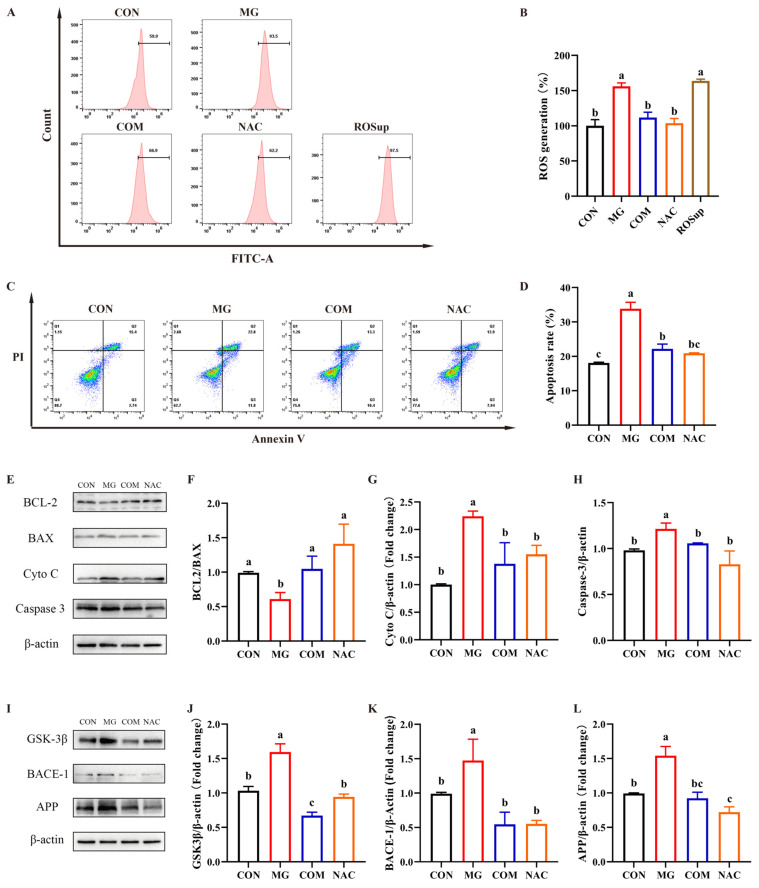
EGCG and metformin inhibited apoptosis and proteins related to Aβ formation by suppressing ROS generation in MG-induced PC-12 cells. COM: 2.5 mM Met + 12.5 μM EGCG. NAC: 10 mM. ROSup: the positive control. (**A**) Analysis of ROS generation by flow cytometry. (**B**) Statistical analysis of the ROS fluorescence intensity. (**C**) Analysis of cell apoptosis by flow cytometry. (**D**) Statistical analysis of the apoptosis rate. (**E**) Western blot results of the expression levels of apoptosis proteins. Statistical analysis of the expression levels of the ratio of Bcl2 and Bax (**F**), cytochrome C (Cyto C) (**G**), and Caspase-3 (**H**). (**I**) Western blot results of the expression levels of proteins related to Aβ formation. Statistical analysis of the expression levels of GSK 3β (**J**), BACE-1 (**K**), and APP (**L**). Data shown are the mean ± SD of three independent experiments. Different letters indicate significant differences between the two groups. (*p* < 0.05).

To further validate that the combination of EGCG and metformin inhibited MG-induced apoptosis in PC-12 cells by suppressing ROS, N-acetylcysteine (NAC) was used as a known ROS scavenger to treat the cells [27]. As shown in Figure 6A,B, ROS generation in MG-treated PC-12 cells were significantly inhibited in both the COM group and NAC group (*p* < 0.05).

A significant inhibition of apoptosis was observed in both the COM group and NAC group in MG-treated PC-12 cells, as demonstrated by Figure 6C,D (*p* < 0.05). Furthermore, as shown in Figure 6E–H, the regulation of BCL2/BAX/Cyto C/caspase 3 expression in the COM group was consistent with that of the NAC group. The expression of BCL2/BAX was enhanced, and the expression of Cyto C and caspase-3 was reduced after treating both the COM and NAC compared to the MG group (*p* < 0.05).

Moreover, NAC was also used to treat the cells to detect the expression of GSK-3β/BACE-1/APP through Western blot. As shown in Figure 6I–L, the expression of GSK-3β, BACE-1, and APP were significantly inhibited in both the COM group and NAC group when compared to the MG group (*p* < 0.05).

## 3. Discussion

Alzheimer’s disease is characterized by neuronal degeneration. Due to its similar pathological features, diabetes is now recognized as an important cause of cognitive dysfunction [28]. The toxicity of methylglyoxal (MG) has been reported to be a critical factor in the development of both diabetes-related cognitive dysfunction and AD. Recently, studies have indicated the new potential of anti-diabetic drugs in the treatment of neurodegenerative diseases, including metformin. However, the side effects still limit its use. Plant-derived drugs are rich in variety and wide in source. Flavonoids are generally regarded as a class of safe compounds and have been used by humans for thousands of years. The safety of the flavonoid has also been proven. For example, quercetin has been recommended at daily doses of 200–1200 mg [29]. The European Food Safety Authority has recommended an EGCG intake of up to 800 mg/day [30]. In this study, the combination of metformin and 0.5% flavonoids (Figure 2. Metformin: Flavonoids = 200:1 in molar) exhibits potential in mitigating MG-induced PC-12 cell damage, and a similar synergistic effect was also observed in SH-SY5Y cells. These data suggested the high efficiency of flavonoids in terms of enhancing the effects of metformin on MG-induced neuronal cell damage.

Flavonoids are chemically composed of two benzene rings (A and B) linked to each other by a hexatomic ring as the typical basic skeleton. They are divided into many subclasses, including flavones, isoflavones, flavonols, flavanols, flavanones, flavan-3-ols, and anthocyanidins, further due to the diverse substituents on the parent nucleus. The SAR of flavonoids combined with metformin was analyzed according to the CI values of MG-induced PC-12 cells viability. The results revealed that the absence of double bonds between the 2 and 3 position, gallate with an R configuration substitution in the 3 position, and ortho-substitution in B ring contributed to exerting the synergistic effect. Our previous study has also revealed that the first two SARs were also relevant to the protective effects of the individual flavonoids on MG-induced PC-12 cells. The third SAR, the ortho-substitution in the B ring was applied and was only relevant to the combination of flavonoids and metformin. These SARs offer a theoretical foundation for the design and modification of flavonoid-based drugs, aiming to enhance cognitive function in individuals with impaired cognition.

The subclass of flavan-3-ols that showed a stronger synergistic effect with metformin, especially the EGCG and low-dose of metformin group, and its mechanism, was explored. Apoptosis has been acknowledged as a programmed cell death process that plays a pivotal role in the pathogenesis of MG-induced neurotoxicity [31]. An extra of MG accumulation enhances ROS production, thereby inducing oxidative stress [32]. This involves the destruction of chemical bonds in biomolecules and leads to the alterations in the structural properties of proteins (enzymes), nucleic acids, and lipids, consequently inducing cellular metabolic dysfunction [33], which is the main trigger for the development of the neurodegenerative disease. As shown in the result (Figure 3), ROS generation increased in PC-12 cells after treatment with MG. An accumulation of ROS leads to mitochondrial damage, resulting in the release of Cyto-C and the subsequent activation of the caspase cascade, ultimately inducing apoptosis [34]. The BCL-2 protein family plays a pivotal role in the mitochondria-mediated apoptotic pathway [35]. BCL-2 plays a pivotal role in promoting cellular survival by exerting its anti-apoptotic efficacy, while Bax counteracts the effect of BCL-2 and promotes apoptosis [36]. Bax can also translocate to the mitochondrial membrane and promote the release of Cyto-C [37].

In addition, BACE-1 is a crucial target for inhibiting the production of β-Amyloid (Aβ) [38]. Furthermore, it was reported that the expression of GSK-3β increased in the brain of AD mice, and its further activation of GSK-3β regulated apoptosis [39]. The previous study demonstrated that the activation of GSK-3β is related to the BACE-1-induced cleavage of APP and Aβ formation [40]. An accumulation of excess Aβ indirectly stimulates the release of reactive oxygen species (ROS), in turn, an overproduction of ROS also promotes the generation of Aβ [41]. Our results demonstrate that the combination of EGCG and metformin inhibited GSK-3β/BACE-1/APP in MG-induced PC-12 cells, while there was no effect when EGCG or 2.5 mM metformin was used alone.

Therefore, our study indicated that the combination of EGCG and metformin effectively inhibited neuronal apoptosis by modulating of the BCL2/BAX/Cyto C/caspase 3 pathway by suppressing ROS generation. Additionally, the combination attenuated MG-induced PC-12 neuronal damage by targeting the signal pathway related to Aβ formation including GSK-3β/BACE-1/APP. Interestingly, while low-dose metformin alone did not exhibit significant regulatory effects on ROS generation and the inhibition of the BCL2/BAX/Cyto C/caspase 3 and GSK-3β/BACE-1/APP pathways, the addition of 0.5% EGCG significantly improved its regulatory effect and achieved a similar efficacy as high-dose metformin, which is three times higher than that of low-dose metformin. These findings were consistent with the results obtained from synergistic index experiments and provided mechanistic insights into the synergistic effect of EGCG on metformin. Additionally, the mechanism study established a robust foundation for the exploration of novel combination therapies aimed at enhancing cognitive function.

However, this study still had certain limitations. For one, more neuroblastoma cell lines, especially, the undifferentiated human monocytic cell line, need be studied to test the synergistic effect of the combination. Furthermore, studies are still needed to verify the synergistic effect of EGCG and metformin and explore the mechanism in vivo in the future.

## 4. Materials and Methods

### 4.1. Reagents

Quercetin (Q), Morin (M), Hesperetin (H), Naringenin (N), (+)-Catechin (C), Epicatechin (EC), Epigallocatechin (EGC), Epicatechin gallate (ECG) and Epigallocatechin gallate (EGCG) were purchased from Sichuan Weikeqi Biological Technology (Chengdu, China) (Purity of all flavonoids used were ≥98%). Metformin (Purity ≥ 98%) was purchased from Solarbio Life Sciences (Beijing, China). Methylglyoxal (MG) was purchased from Aladdin (Shanghai, China). Antibodies against β-actin, was purchased from Proteintech (Wuhan, China). Antibodies against GSK-3β and APP was purchased from Abclonal (Wuhan, China). Antibodies against BACE-1 was purchased from Beyotime Biotechnology (Beijing, China). The secondary anti-rabbit HRP-conjugated antibodies were purchased from Abclonal (Wuhan, China). NAC (Purity ≥ 98%) was purchased from MedChemExpress (MCE, Shanghai, China).

### 4.2. Cell Culture and Treatment

Rat pheochromocytoma PC-12 cells and human neuroblastoma SH-SY5Y cells were obtained by Chinese Academy of Sciences Cell Bank (Shanghai, China) and cultured in RPMI 1640 (Gibco, NH, USA) supplemented with 10% fetal bovine serum (FBS), and 100 U/mL penicillin and 100 g/mL streptomycin in a humidified 5% CO_2_ atmosphere at 37 °C.

### 4.3. Cell Counting Kit-8 (CCK-8) Assay

Cell viability was detected by CCK-8 assay. PC-12 cells were seeded into 96-well plates (1 × 10^4^ cells/well) overnight and were treated with flavonoids and metformin co-cultured with MG. SH-SY5Y cells (refraining from any inducement to differentiate) were treated with EGCG and metformin co-cultured with MG. At the end of the incubation for 48 h, 10 μL CCK-8 reagent (APE×bio, Houston, TX, USA) was added into each well, and cells were incubated for another 2 h at 37 °C. The absorbance at 450 nm was measured by a microplate reader (SpectraMax M2e, Molecular Device, San Jose, CA, USA).

### 4.4. Combination Index (CI) Calculation

To illustrate the interaction between the flavonoids and metformin quantificationally, the combination index (CI) was calculated according to Chou and Talalay’s method based on the median-effect principle [25].

The equation for the median-effect principle is as follows:


(1) log⁡D=log⁡Dm+1mlog⁡fa∕fu

fa is the fraction affected by dose D, fu is the unaffected fraction (fu = 1 − fa), m is the coefficient signifying the shape of the dose–effect curve, D is the dose of the agonist, and Dm is the median-effect dose (EC_50_ in this article [42]). As the values have to be between 0 and 1, they were normalized to the highest value of the dataset and not to the solvent control, as well as multiplied by 0.99 for further analysis [43].

The equation for the CI is as follows:

(2) $$CI=\frac{{\left(D\right)}_{1}}{{\left(Dx\right)}_{1}}+\frac{{\left(D\right)}_{2}}{{\left(Dx\right)}_{2}}$$

(*D*)_1_ and (*D*)_2_ are the concentrations of compounds that work x% effect in a combination system, and (*Dx*)_1_ and (*Dx*)_2_ are the concentrations of each compound alone causing *x*% effect. The combined inhibition was divided into synergism (*CI* < 0.9), additive effect (*CI* = 0.9−1.1), or antagonism (*CI* > 1.1), according to the *CI* values.

### 4.5. Intracellular Reactive Oxygen Species (ROS) Assay

Intracellular ROS was detected using the fluorescence probe DCFH-DA (Beyotime Biotechnology, Shanghai), which has no fluorescence until it is oxidized following permeation into the cell membrane. The level of intracellular ROS can be quantified by the measurement of the fluorescence intensity of oxidized DCFH-DA. PC-12 cells were seeded into 60 mm plates overnight and treated with 10 mM metformin as a conventional dose and 2.5 mM as a low dose, and EGCG (12.5 μM), respectively, and co-treated with 2.5 mM metformin and 12.5 μM EGCG as the combination group. Cells were pre-treated by NAC for 2 h. All groups were co-cultured with 0.5 mM MG for 48 h. After treatment for 48 h, the PC-12 cells were collected and washed three times with PBS. The positive control group was treated by ROSup for 30 min. Then, cells were incubated with 10 μM DCFH-DA at 37 °C for 30 min in the dark and washed three times with serum-free RPMI 1640. The level of ROS was detected by flow cytometry (BD Biosciences, Bergen, NJ, USA).

### 4.6. Cell Apoptosis Analysis

Cell apoptosis was detected by flow cytometry (BD Accuri C6, Franklin-Lakes, NJ, USA), wherein PC-12 cells were placed in 60 mm plates, the treatment was same as 4.5. Additionally, after the treatment, cells were stained with PI and Annexin V-fluorescein isothiocyanate (FITC) according to manufacturer’s protocols (Elabscience Annexin V-FITC/PI Apoptosis Kit, Wuhan, China). The apoptotic PC-12 cells were analyzed with FlowJo_V10.8.1.

### 4.7. Western Blotting

Cells were treated the same as 4.5. and then washed twice with ice-cold PBS and were lysed in RIPA buffer (Beyotime Biotechnology, Shanghai, China) after the treatment with metformin or/and EGCG co-cultured with MG. The Supernatant was collected, and concentration was determined by BCA protein assay kit (Solarbio, Beijing, China). The same amounts of proteins were separated by sodium dodecyl sulfate polyacrylamide gel electrophoresis (SDS-PAGE) and transferred onto PVDF membranes (Merck Millipore, Darmstadt, Germany). The membranes were blocked with 5% skim milk for 2 h at room temperature, followed by overnight incubation at 4 °C with primary antibodies Bcl-2 (12789-1-AP, Proteintech), Bax (50599-2-Ig, Proteintech), Caspase-3 (A11040, ABclonal), Cytochrome C (A4912, ABclonal), GSK-3β (A2081, ABclonal), BACE-1 (AF6273, Beyotime), APP (A17911, ABclonal), and β-actin (AC038, ABclonal). The membranes were washed with 1% TBST three times, and then incubated with secondary antibodies (1:3000 dilution) for 1 h at room temperature. Finally, the bands were visualized using an ECL kit (Tanon, Shanghai, China).

### 4.8. Statistical Analysis

All experiments were performed three times. Data are presented as mean ± standard deviation (SD). SPSS 22.0 software was used for statistical analysis, and the significant difference was determined by one-way analysis of variance (ANOVA). *p* < 0.05 was considered statistically significant.

## 5. Conclusions

In this study, flavonoids were found to protect PC-12 cells from MG-induced damage with metformin synergistically which provided an approach to alleviating neurodegenerative diseases. The SAR analyze could also supply a theoretical basis for the design of the combination. The mechanistic study demonstrated that the typical flavonoids, EGCG, enhanced protective activities via ROS-mediated apoptosis and the pathway related to amyloid plaques (Aβ) formation. The data from this research suggest that the combination of flavonoids with metformin has the potential to be developed into alternative means for the treatment or amelioration of the condition of neurodegenerative disease by this target.

## Data Availability

Data are contained within the article and Supplementary Materials.

## Supplementary Materials

The following supporting information can be downloaded at: https://www.mdpi.com/article/10.3390/molecules29102306/s1, Figure S1: Cell viability of drug combinations on PC-12 cells. Figure S2: Protective effect of the combination of metformin and EGCG against MG-induced SH-SY5Y cells.
