# Supplementary material for: Synergistic Effect of Flavonoids and Metformin on Protection of the Methylglyoxal-Induced Damage in PC-12 Neuroblastoma Cells: Structure–Activity Relationship and Potential Target
Source: Molecules. 2024 May 14;29(10):2306. doi: 10.3390/molecules29102306 (PMC11124423; doi:10.3390/molecules29102306)
Supplement: Supplementary file 1 [file molecules-29-02306-s001.zip › molecules-2904821-supplementary.pdf]

Supplementary Information

Synergistic effect of Flavonoids and Metformin on protection of the Methylglyoxal-Induced Damage in PC-12 Neuroblastoma Cells: Structure-Activity Relationship and Potential Target

Danyang Zhang [1], Xiaoshi He [1], Ting Wang [2], Yan Xing [1], Jing Wang [2], Zhilong Xiu [1], Yongming Bao [1, 3], Zhansheng Zhao [2*], Yuesheng Dong [1*]

1 MOE Key Laboratory of Bio-Intelligent Manufacturing, School of Bioengineering, Dalian University of Technology, Dalian, 116024, China

2 Department of Endocrinology, The Second Hospital of Hebei Medical University, Shijiazhuang, Hebei, 050000, China

3 School of Ocean Science and Technology, Dalian University of Technology, Panjin, 124221, China

* To whom correspondence should be addressed:

Zhansheng Zhao, Department of Endocrinology, The Second Hospital of Hebei Medical University, No.215 Heping West Road, Shijiazhuang, 050000, China.

Email: zhshzhao@sina.com

Yuesheng Dong, School of Bioengineering, Dalian University of Technology. No. 2 Linggong Road, Dalian, 116024, China.

E-mail: yshdong@dlut.edu.cn

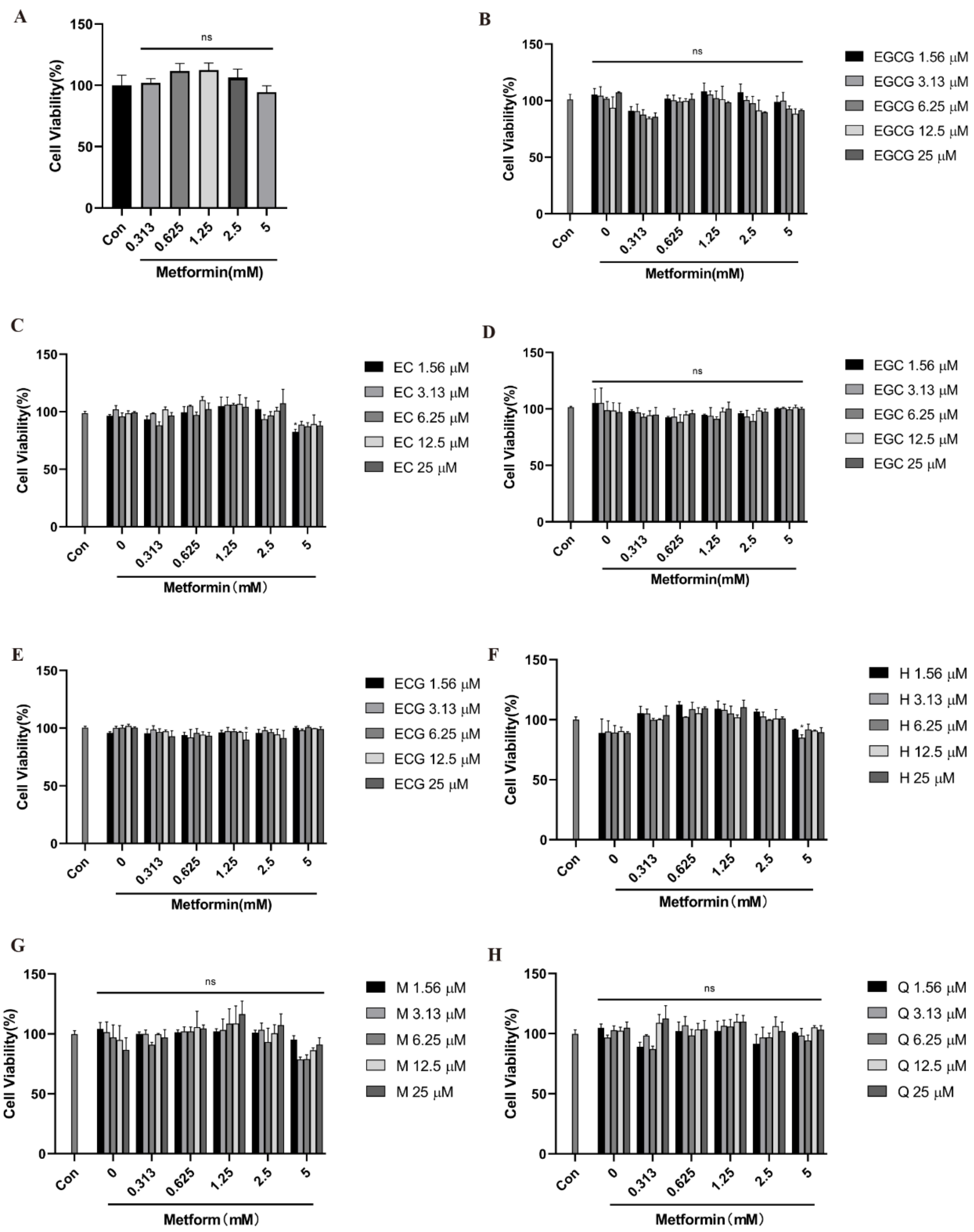

Figure S1 Cell viability of drug combinations on PC-12 cells. Data shown are the mean ± SD of three independent experiments. n.s. indicate no significant differences between the two groups. (*p*>0.05)

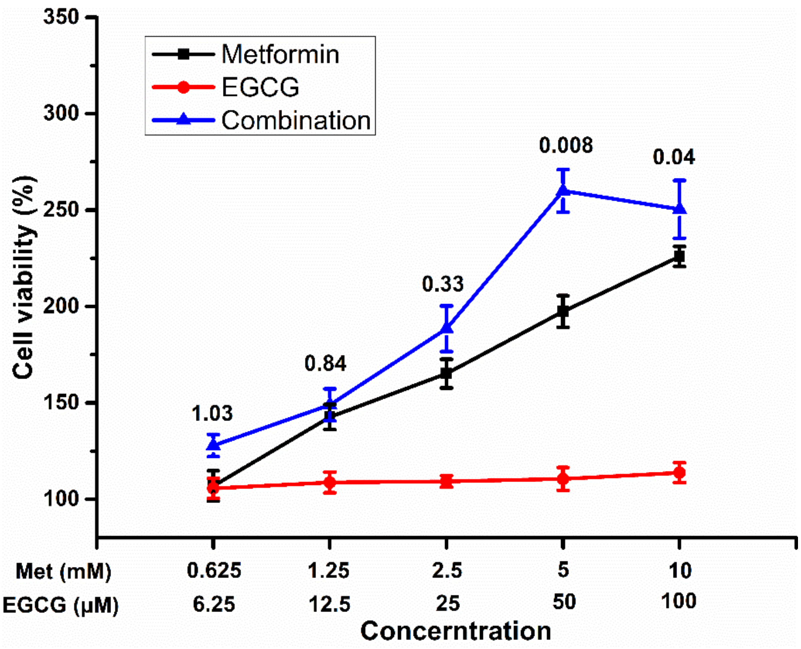

Figure S2 Protective effect of the combination of metformin and EGCG against MG-induced SH-SY5Y cells.
